# Mental Balance in 116 Nations: Where It Is Experienced and Valued

**DOI:** 10.3390/ijerph191912457

**Published:** 2022-09-30

**Authors:** Mohsen Joshanloo

**Affiliations:** Department of Psychology, Keimyung University, 1095 Dalgubeol Boulevard, Dalseo-gu, Daegu 42601, Korea; mjoshanloo@kmu.ac.kr

**Keywords:** mental balance, culture, global, national wealth, peace, Gallup

## Abstract

Mental balance, defined as a sense of tranquility resulting from inner peace and harmonious interactions with the external environment, is an important but largely overlooked aspect of well-being. Using data from the Gallup World Poll (N = 121,207), this study developed a global index of mental balance and a measure of preference for mental balance (as opposed to excitement) across 116 countries. The study examined the global and regional distribution of these two variables and their intercorrelations with a variety of social, economic, cultural, and well-being variables. The results showed that, whereas national wealth and sociopolitical context were the strongest predictors of experiencing mental balance, these variables were not associated with preference for mental balance.

## 1. Introduction

Mental balance can be defined as a sense of tranquility that arises from inner peace and harmonious relationships with the external world. A sense of mental balance is experienced when a person feels that the various aspects of their lives are in balance, and they feel at peace with life. Calmness, relaxation, and tranquility are examples of low-arousal feelings that indicate a sense of mental balance and harmony. Mental balance and harmony are multi-faceted constructs. Delle Fave et al. [1] suggested that harmony can be experienced and studied at three levels: the intrapersonal level (inner harmony), the interpersonal level (harmonious social relations), and the transcendental level (harmonious relations with broader natural or spiritual elements).

Concepts related to mental balance and harmony are largely absent from prevailing Western models of well-being [1]. Instead, life satisfaction has been the primary focus of global well-being studies [2,3]. Positive and negative affect and psychosocial functioning have also recently attracted some attention [4,5]. However, mental balance and harmony as aspects of well-being have been largely overlooked in global studies. Concepts related to balance and harmony (e.g., work–life balance research and low-arousal emotions) have received more attention in organizational and cultural psychology [6,7]. In the recent decade, however, well-being researchers have placed a greater emphasis on mental balance as a crucial but unappreciated component of mental health, advocating for additional research in this area [1,8]. This interest is partly due to the fact that qualitative studies examining lay definitions of well-being in different countries have shown that people often refer to harmony and balance as crucial components of well-being [9]. Accordingly, measurement instruments have been developed to facilitate the empirical exploration of mental balance and harmony, such as the peace of mind scale [10] and the harmony in life scale [11]. Research has shown that mental balance and harmony are associated with a variety of desirable outcomes [8,11]. What has been lacking are large-scale multinational studies of mental balance and harmony to examine their global distribution. The present study aimed to fill this gap.

In 2020, the Gallup World Poll (GWP) included three questions related to mental balance in its global survey. The survey also included a question assessing preference for a calm (versus an exciting) life, which can be used as a measure of the value placed on mental balance by individuals and cultures. Thus, it is now possible to study mental balance and the desire for it as an aspect of national well-being. Using the GWP, the present country-level study had two objectives: 1—to draw a global picture of the state of mental balance and preference for it, and 2—to establish the nomological networks of these variables by looking at their associations with other well-being, social, economic, and cultural variables.

## 2. Methods

### 2.1. Participants

The mental balance questions were included in the GWP 2020, which was conducted mostly in 2020 but also early 2021 in 116 countries. A total of 121,207 people took part in the survey (average age = 39.812, *SD* = 16.696, females = 48.9%). For more information about the sample, descriptive information, and preliminary results on the items used in this study, see [12]. The GWP conducts nationally representative surveys in more than 100 countries. Typically, these surveys are conducted annually in each country by random digit dialing (RDD) or face-to-face interviewing [13]. The average sample size in each country is about 1000 people per year, but larger samples are collected in countries with large populations (e.g., China). Specific information about data collection dates and modes in each country can be found at https://www.gallup.com/services/177797/country-data-set-details.aspx (accessed on 21 September 2022).

### 2.2. Measures

**Mental balance and preference for it.** Using a yes/no response format, respondents were asked whether they felt calm during a lot of the day yesterday, whether they felt at peace with their lives, and whether they felt that the various aspects of their lives were in balance. Preference for a calm life was calculated as the percentage of people in a country who chose “a calm life” in response to the question, “Would you rather live an exciting life or a calm life?”. Other response options were “an exciting life”, “both”, and “neither”. 

**GWP-based variables**. Some GWP-based well-being indices were calculated based on Helliwell et al. [2] and Joshanloo [4]. To assess life satisfaction, participants were asked which step of the life ladder they thought they were on, from 0 = *worst possible* to 10 = *best possible*. Future life satisfaction was assessed by asking on which step they felt they would be “in the future, say about five years from now?”. Positive affect was measured with two items asking respondents if they felt enjoyment for much of the day yesterday and if they smiled or laughed a lot yesterday. The negative affect measure consisted of four questions asking the participants if they felt worry, sadness, stress, and anger during much of the day yesterday. The eudaimonic index [4] was used to measure optimal psychosocial functioning. This index is composed of seven GWP items that measure learning experiences, social support, respect, beliefs about efficacy, sense of freedom, and social interest. However, the item related to efficacy was not included in 2020 and was therefore excluded. The perceived injustice index [14] is based on four of the GWP items that ask participants about their perceptions of local police, efforts to help the poor, the justice system and courts, and respectful and dignified treatment of women in their city/country. Religiosity was measured by calculating the proportion of individuals in each country who reported that religion was an important part of their lives [15].

**Cultural Dimensions**. Hofstede’s [16] main cultural dimensions were included: individualism/collectivism (the strength of one’s ties to one’s ingroups), power distance (the degree of power inequality accepted between people), masculinity (the degree to which men’s and women’s roles do not overlap), and uncertainty avoidance (the degree to which uncertainty is accepted).

**Social progress**. Social progress is defined as “the capacity of a society to meet the basic human needs of its citizens, establish the building blocks that allow citizens and communities to enhance and sustain the quality of their lives, and create the conditions for all individuals to reach their full potential” ([17], p. 3). The social progress index excludes financial aspects of progress and instead identifies the social and environmental elements of countries’ performance. It consists of three main dimensions [17]: basic human needs (how well a country meets the basic needs of its citizens, such as nutrition and basic health care, water and sanitation, shelter, and personal safety), foundations of well-being (whether citizens have free access to basic education and domestic and foreign information, as well as health and environmental quality), and opportunity (the extent to which a country’s citizens have personal rights and freedoms and access to advanced education, as well as the extent of social inclusion). This study uses the overall social progress index and its three sub-indices.

**Income inequality**. Income inequality was measured using the World Bank’s estimated Gini index (https://data.worldbank.org/indicator/SI.POV.GINI, accessed on 11 November 2021). All of the countries’ ratings from 2016 to 2019 were averaged to reduce missing values as much as possible.

**National wealth**. GDP per capita (Purchasing Power Parity, current international dollar) 2019 was used to measure national wealth (data.worldbank.org/indicator/NY.GDP.PCAP.PP.CD, accessed on 11 November 2021). This variable was log-transformed.

**Ecological stress**. Ecological stress from extreme heat, extreme cold, pathogens, inland topography, and mountain topography was measured using Conway et al.’s [18] index of cumulative ecological stress. 

**Peace**. The state of peace in three domains of societal safety and security, ongoing domestic and international conflict, and militarization was measured using the global peace index 2020 (www.visionofhumanity.org, accessed on 11 November 2021). The variable was reversed such that higher scores indicate more peacefulness.

**National age.** National age was measured by calculating the proportion of the total population in each country aged 65 and above using the World Bank data (data.worldbank.org/indicator/SP.POP.65UP.TO.ZS, accessed on 11 November 2021).

**Human development index.** The human development index 2020 (hdr.undp.org, accessed on 21 September 2022) was used as a measure of average performance on three key dimensions of human development: a long and healthy life, knowledge acquisition, and an adequate standard of living. The three dimensions are assessed by life expectancy at birth, years of schooling for adults 25 years and older as well as expected years of schooling for children of school entry age, and gross national income per capita. 

## 3. Results

### 3.1. Developing National Indices: Factor and Reliability Analyses

**Figure 1:**
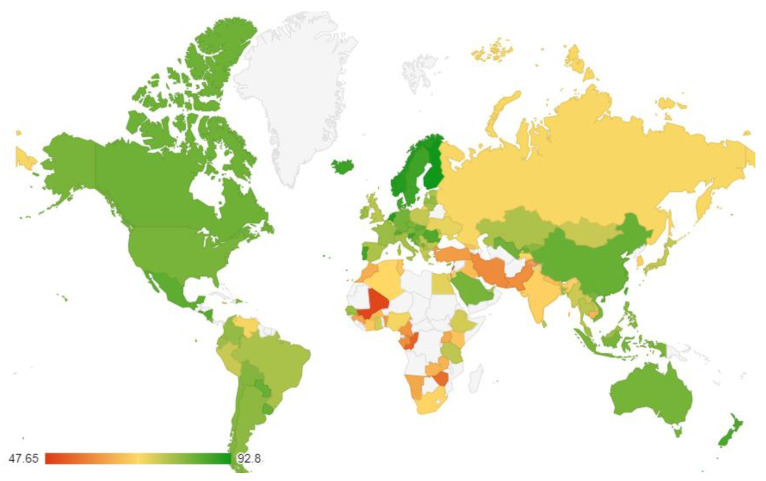
Balance index around the world. The grey color indicates no available data.

Three national variables were calculated, indicating the percentage of individuals in each country who answered “yes” to the three mental balance questions. These three variables were used in a principal component analysis at the country level. The results confirmed that the data for the three variables had a single-factor structure. A component with an eigenvalue of 2.05 explained about 69% of the variance in the three items. Factor loadings were 0.915, 0.654, and 0.890 for balance, calm, and peace, respectively. The Cronbach’s alpha was 0.763, suggesting an acceptable internal consistency. An index of mental balance was developed by averaging the three variables per country (*M* = 76.225, *SD* = 9.179, Min = 47.65, Max = 92.80). The global status of mental balance is depicted in Figure 1. Tables S1 and S2 in the Supplementary Materials provide country scores and rankings. 

**Figure 2:**
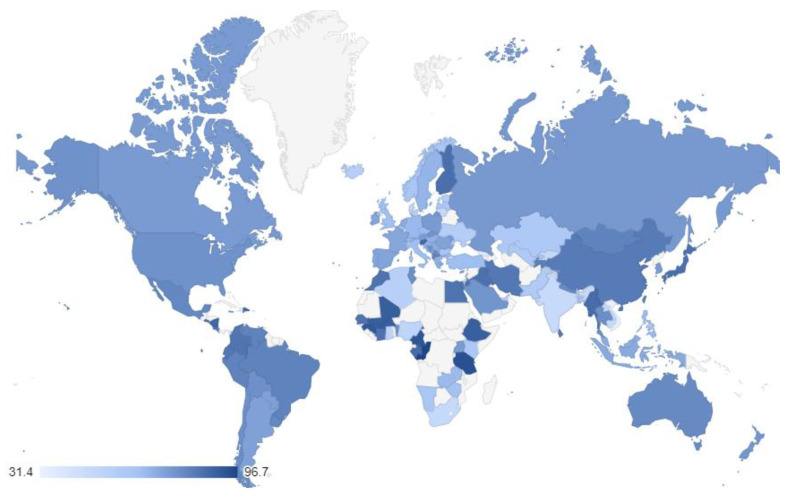
Preference for a calm life around the world. The grey color indicates no available data.

The percentage of individuals per country that chose a calm life are shown in Tables S1 and S3 and Figure 2 (*M* = 72.381%, *SD* = 12.805, Min = 31.4, Max = 96.7). 

### 3.2. Regional Status

**Figure 3:**
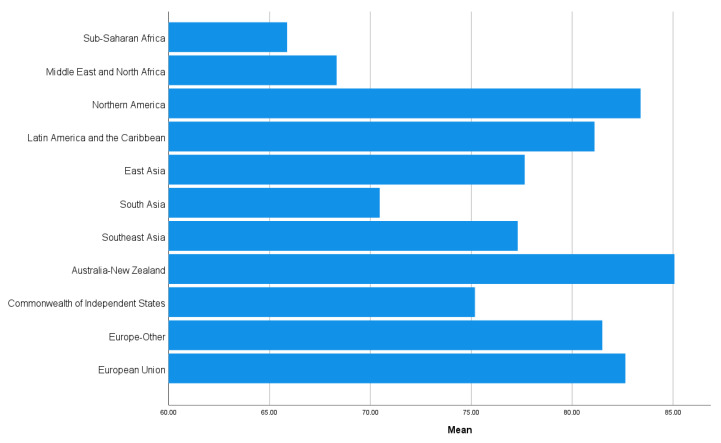
Regional means of experienced mental balance.

**Figure 4:**
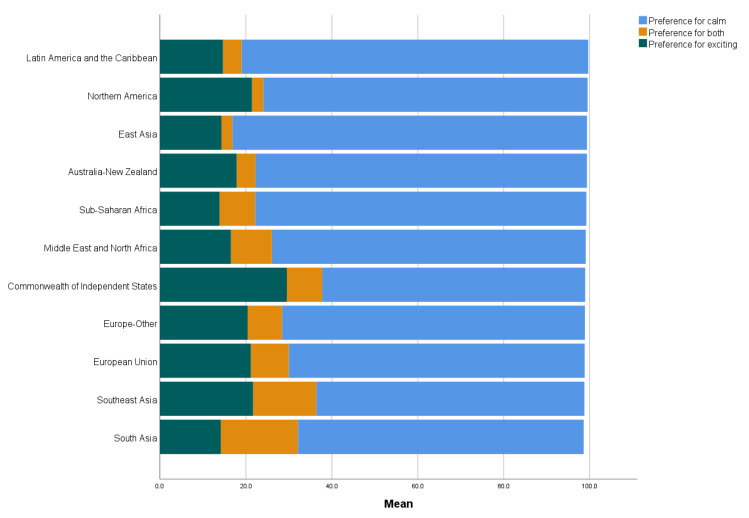
Regional means of preference for a calm life.

Figure 3 and Figure 4 show the regional means of mental balance and preference for a calm life. Wealthier regions had a higher experienced mental balance. For calmness preference, East Asia and Latin America and the Caribbean had the highest scores, whereas the commonwealth of independent states (including ex-Soviet countries) had the lowest score. The list of countries belonging to each region is given in Table S1. 

### 3.3. Nomological Networks

Mental balance and preference for a calm life were not significantly correlated (*r* = −0.052). The correlations with other variables are reported in Table 1. Only correlations above 0.20 are considered nontrivial and discussed. 

**Figure 5:**
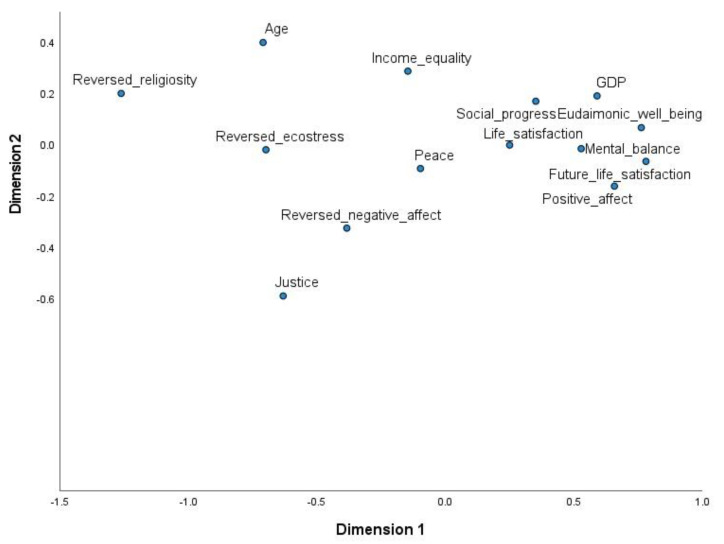
Multidimensional scaling plot.

**Mental balance**. Among the well-being variables, life satisfaction correlated most strongly with mental balance, while the other correlations were moderate. Among the cultural dimensions, mental balance was negatively and moderately correlated with religiosity. There was also a weak positive correlation with individualism. In the category of socio-economic–ecological variables, the strongest correlations were with social progress dimensions, human development, GDP, peace, and national age. Mental balance was also moderately and negatively correlated with income inequality, ecological stress, and perceived injustice. Given the strong correlations between the variables (indicating multi-collinearity), regression analysis could not be used to examine the relative importance of variables in explaining the variance in mental balance. Hence, multi-dimensional scaling was used to create a two-dimensional map, containing only variables with moderate to high correlations with mental balance. Due to the large overlap with GDP and social progress, the human development index was not included in this analysis. For this analysis, variables with a negative association with mental balance were reversed (e.g., negative affect was transformed to show the absence of negative affect), and all variables were rescaled to range between −1 and 1. The resulting plot is shown in Figure 5. Variables closer to mental balance are those with the strongest associations with mental balance after controlling for all inter-relations. Some well-being variables (i.e., eudaimonic well-being, life satisfaction, positive affect, and future life satisfaction) along with GDP and social progress are the best signifiers of experienced mental balance at the national level. 

**Preference for a clam life**. Preference for a calm life was unrelated to most of the variables. There were weak but nontrivial correlations with negative affect (+), individualism (−), perceived injustice (+), masculinity (+), and uncertainty avoidance (+). 

### 3.4. Supplementary Analysis: Relationship with Age and Gender

**Figure 6:**
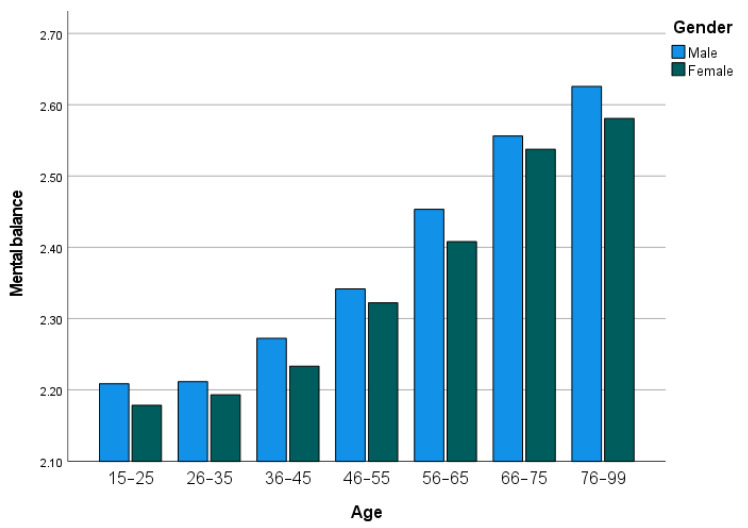
Mental balance across age and gender groups.

As can be seen in Figure 6, mental balance is positively associated with age at the individual level. Men scored slightly higher than women of all ages, but the gender difference was too small across the whole sample, with a small effect size of 0.021. Individuals who chose a calm life were on average older (*M* = 41.320) than individuals who chose other options (*M* = 35.892). The *t*-value (53.391) was significant at *p* < 0.001, with a medium effect size of 0.329. Of males, 69.6%, and of females, 75.0% chose a calm life over the other options, indicating that females placed more value on calmness.

## 4. Discussion

### 4.1. Mental Balance

The three mental balance items are highly consistent at the national level, and the data support a one-factor structure. Mental balance is higher in affluent regions (e.g., Australia and New Zealand, North America, and the European Union) and lower in less wealthy regions (e.g., Africa, the Middle East, and South Asia). Mental balance is strongly correlated with national life satisfaction (Table 1), which in turn is strongly linked to national wealth (for a review and empirical evidence, see [5]. It can be concluded that mental balance and life satisfaction are closely related to markers of economic and political development (at the national level). In contrast, other well-being variables (e.g., eudaimonic well-being, and positive and negative affect) are more independent of national wealth [5]. The patterns of associations between mental balance and other variables are consistent with this conclusion (Table 1). Notably, the association was stronger with positive affect than with negative affect, potentially reflecting the fact that mental balance is itself a positive subjective state. Mental balance is highly associated with social progress, national prosperity, and peacefulness. Culturally, mental balance is associated with lower levels of religiosity and higher levels of individualism. The results of multidimensional scaling imply that cultural and demographic factors (such as individualism, religiosity, and national age) become less influential when wealth and peacefulness are taken into account. It can be concluded that stability, prosperity, and socio-political development are crucial conditions for a high national level of mental balance.

### 4.2. Preference for Calm Life

The preference for mental balance was highest in East Asia, which is in keeping with numerous studies showing that East Asian countries favor low-arousal emotions more than Western countries [19]. Daoism, Buddhism, and Confucianism, among other East Asian systems of thought, believe that a sense of mental balance and harmony is essential to human well-being [20], a notion that may underlie these cultural differences. Clearly, there was no correspondence between national or regional wealth and preference for a calm life. In the absence of correlations with most of the variables included, it is challenging to explain the global distribution of calmness preference. Some weak but nontrivial correlations suggest that people who live in countries with higher levels of collectivism, masculinity, uncertainty avoidance, negative affect, and subjective injustice are more likely to prefer a calm life to a life of excitement. Psychological and cultural dimensions thus play a greater role than socioeconomic conditions in determining the preference for calmness. Given the weak associations, however, it seems fair to conclude that, at this stage, we do not know much about the factors that determine the global distribution of calmness preference and that further studies using alternative measures of this concept are needed.

### 4.3. Age and Gender Differences

Consistent with the results of prior research [21,22], older people were more likely to experience and desire mental balance. In addition, women were found to prefer a calm (as opposed to an exciting) life more than men, which is consistent with the findings of [23]. 

## 5. Conclusions

This study examined experienced mental balance and desire for a calm life on a global scale. The two factors were unrelated and had largely different nomological networks, suggesting that, at the national level, the experience and value of mental balance are distinct concepts. While peace, prosperity, and socio-political development are critical to high levels of mental balance in a country, psychological and cultural factors play a greater role in determining the desire for a calm life. The correlations between mental balance and other variables of well-being ranged from 0.395 to 0.757, which can be taken as an indication of convergent validity for the new index of metal balance. This also suggests that mental balance has unique variance and captures aspects of national well-being that are not captured by other variables of well-being. Future studies need to examine experience and value of mental balance and their nomological networks at the individual level as well. It should also be noted that the data were collected during the COVID 19 pandemic, which may have influenced the results. However, in the absence of comparable pre-pandemic data, the magnitude of the impact of the pandemic on the results cannot be quantified. One global study found that, contrary to the expectations of many, the pandemic did not have a large impact on national life satisfaction in 2020 [2]. In some countries, subjective well-being improved slightly or remained unchanged in 2020. Thus, although the severe hardship caused by the pandemic significantly affected many people’s lives, we cannot determine with certainty the extent to which these effects are reflected in national mental balance scores in 2020. A fruitful avenue for future research would be to compare mental balance scores in 2020 with those in later years (if available), when the effects of the pandemic are subsiding.

## Figures and Tables

**Table 1:** Correlations between mental balance and preference for calm life and other national variables.

		Mental Balance		Preference for a Calm Life	
		*r*	N	*r*	N
Well-being					
	Eudaimonic well-being	0.522 ***	116	−0.115	116
	Negative affect	−0.455 ***	115	0.251 **	115
	Positive affect	0.575 ***	116	−0.094	116
	Life satisfaction	0.757 ***	116	−0.180	116
	Future life satisfaction	0.395 ***	116	−0.172	116
Cultural dimensions					
	Individualism	0.238	63	−0.243	63
	Power distance	−0.168	63	0.174	63
	Masculinity	−0.173	63	0.214	63
	Uncertainty avoidance	−0.126	63	0.219	63
	Religiosity	−0.514 ***	107	0.178	107
Socio-economic-ecological variables					
	Income inequality	−0.383 ***	89	0.169	89
	Ecological stress	−0.398 ***	113	0.080	113
	GDP	0.592 ***	114	−0.147	114
	Peace	0.608 ***	114	−0.101	114
	Perceived injustice	−0.327 ***	116	0.215 *	116
	Population ages 65 and above	0.531 ***	114	−0.124	114
	Social progress	0.637 ***	112	−0.177	112
	Basic human needs	0.618 ***	112	−0.166	112
	Foundations of wellbeing	0.631 ***	112	−0.166	112
	Opportunity	0.564 ***	112	−0.169	112
	Human development	0.621 ***	112	−0.199 *	112

Note. *** *p* < 0.001. ** *p* < 0.01. * *p* < 0.05.

## Data Availability

All data used in this study are publicly available from original sources, with the exception of Gallup data, which can be purchased from Gallup. For more information, see https://www.gallup.com/analytics/318875/global-research.aspx (accessed on 1 November 2021). Variables calculated using the Gallup World Poll are provided in Tables S1–S3 in the Supplementary Materials.

## Supplementary Materials

The following supporting information can be downloaded at: https://www.mdpi.com/article/10.3390/ijerph191912457/s1, Table S1: Descriptive Information; Table S2: Country Ranking for Mental Balance; Table S3: Country Ranking for Preference for Calm Life.
