# Supplementary material for: Mental Balance in 116 Nations: Where It Is Experienced and Valued
Source: Int J Environ Res Public Health. 2022 Sep 30;19(19):12457. doi: 10.3390/ijerph191912457 (PMC9564841; doi:10.3390/ijerph191912457)
Supplement: Supplementary file 1 [file ijerph-19-12457-s001.zip › ijerph-1929085-supplementary.pdf]

Table S1
Descriptive Information

Region	Country	N	Mental Balance	Would you rather live an exciting life or a calm life?		
				Calm	Exciting	Both
European Union	Austria	1000	82.17	74.0	16.5	8.2
	Belgium	1005	77.38	64.0	18.6	17.2
	Bulgaria	1000	76.43	62.8	29.6	7.3
	Croatia	1002	83.73	66.9	20.8	11.0
	Cyprus	1005	80.93	74.3	17.8	7.1
	Czech Republic	1004	82.90	65.4	17.7	15.1
	Denmark	1000	88.00	52.1	40.8	6.8
	Estonia	1000	77.87	56.8	22.2	20.5
	Finland	1000	92.80	85.7	11.4	2.8
	France	1000	79.63	71.3	21.0	5.9
	Germany	1000	83.30	66.5	23.5	7.3
	Greece	1002	75.68	68.4	26.9	3.4
	Hungary	1001	83.85	68.7	20.8	10.3
	Ireland	1000	79.50	71.4	19.2	7.8
	Italy	1000	78.93	65.1	30.0	3.7
	Latvia	1001	80.25	60.9	31.0	7.2
	Lithuania	1002	78.68	48.1	14.8	32.4
	Malta	1001	85.98	77.7	16.9	5.2
	Netherlands	1006	90.66	69.3	27.0	2.8
	Poland	1010	75.87	74.2	20.8	3.9
	Portugal	1002	88.06	71.2	19.4	8.6
	Romania	1000	86.70	72.3	15.2	10.9
	Slovakia	1001	84.68	75.6	15.4	8.0
	Slovenia	1001	88.15	87.5	8.7	3.3
	Spain	1000	78.03	71.9	18.0	7.6
	Sweden	1000	88.47	67.6	26.0	5.7
Europe-Other	Albania	1000	74.90	80.4	10.8	8.0
	Bosnia Herzegovina	1001	81.95	77.4	17.5	4.2
	Iceland	501	88.62	55.1	31.3	11.2
	Kosovo	1000	82.43	77.1	16.8	5.9
	Montenegro	1004	79.02	72.0	18.2	8.9
	North Macedonia	1003	77.83	79.5	10.9	9.0
	Norway	1000	91.00	61.5	30.9	7.1
	Serbia	1002	76.35	69.5	19.7	9.1
	Switzerland	1000	86.23	67.7	22.4	8.3
	United Kingdom	1000	76.63	64.5	25.6	8.5
Commonwealth of Independent States	Georgia	1003	67.20	36.0	61.4	2.6
	Kazakhstan	1000	77.97	58.9	22.5	14.5
	Kyrgyzstan	1000	81.37	48.3	30.2	21.3
	Moldova	1000	78.13	74.8	21.1	3.4
	Russia	2022	70.90	74.7	22.7	1.9
	Tajikistan	1000	70.07	81.3	9.7	8.6

	Ukraine	1001	72.26	55.9	35.3	7.4
	Uzbekistan	1000	83.57	59.5	33.4	6.0
Australia-New Zealand	Australia	1003	83.12	78.7	16.7	4.0
	New Zealand	1002	87.03	75.5	19.0	4.8
Southeast Asia	Cambodia	1002	65.34	47.8	22.0	27.8
	Indonesia	1062	82.89	70.2	25.1	3.8
	Laos	1000	74.87	50.6	15.8	30.7
	Malaysia	1000	79.27	69.6	19.2	10.8
	Myanmar	1000	75.17	86.4	13.0	.5
	Philippines	1000	81.53	65.7	21.5	12.7
	Thailand	1000	76.70	77.5	13.4	8.5
	Vietnam	1000	82.67	31.4	43.4	22.8
South Asia	Bangladesh	1013	80.16	65.5	11.1	20.6
	India	3103	68.99	47.1	23.3	27.0
	Nepal	1000	66.53	78.8	16.9	4.1
	Pakistan	1000	59.80	57.9	8.3	33.2
	Sri Lanka	1000	76.83	83.2	11.3	4.7
East Asia	China	3503	84.45	82.5	12.0	4.0
	Hong Kong	1005	73.30	91.0	8.2	.5
	Japan	1016	76.02	85.4	11.6	1.9
	Mongolia	1000	75.53	80.5	17.5	2.0
	South Korea	1005	69.25	74.4	23.4	1.7
	Taiwan	1000	87.33	81.1	13.2	5.3
Latin America and the Caribbean	Argentina	1000	80.97	72.7	22.1	4.5
	Bolivia	1001	81.85	77.1	18.6	4.0
	Brazil	1002	78.28	80.5	17.3	1.9
	Chile	1000	81.80	77.0	17.8	4.9
	Colombia	1000	80.30	84.2	14.5	1.3
	Costa Rica	1000	82.80	82.6	13.6	3.5
	Dominican Republic	1000	81.83	87.8	9.9	2.1
	Ecuador	1000	79.50	83.5	12.7	3.6
	El Salvador	1000	86.70	85.3	11.9	2.5
	Jamaica	502	76.76	78.9	8.6	12.0
	Mexico	1010	85.38	79.3	14.3	5.7
	Nicaragua	1002	86.03	88.4	8.3	3.0
	Paraguay	1000	84.47	73.0	14.3	12.2
	Peru	1006	75.55	80.4	17.6	1.8
	Uruguay	1001	84.38	79.3	18.3	2.0
	Venezuela	1000	71.17	80.4	14.9	4.4
Northern America	Canada	1006	83.83	74.3	22.4	3.1
	United States	1007	82.95	76.7	20.5	2.2
Middle East and North Africa	Algeria	1016	69.95	54.8	13.2	30.8
	Bahrain	1009	82.42	74.5	19.6	5.2
	Egypt	1002	72.89	84.5	10.9	4.2
	Iran	1009	59.20	83.4	15.1	1.3
	Iraq	1000	66.20	85.2	7.0	7.4
	Israel	1057	63.20	51.5	24.6	21.9

	Jordan	1012	72.04	84.3	10.4	5.2
	Lebanon	1050	47.65	69.8	8.0	21.0
	Morocco	1006	64.21	84.8	7.3	6.0
	Saudi Arabia	1043	82.97	75.8	19.1	5.0
	Tunisia	1003	67.50	77.5	18.7	2.7
	Turkey	1000	61.70	64.9	28.0	5.7
	United Arab Emirates	1011	78.34	58.0	32.9	7.8
Sub-Saharan Africa	Benin	1042	60.52	78.0	2.8	17.3
	Burkina Faso	1007	65.94	90.2	6.7	2.6
	Cameroon	1000	60.53	92.3	6.1	1.0
	Congo Brazzaville	1002	53.46	96.7	1.9	.7
	Ethiopia	1003	74.44	89.7	8.9	1.3
	Gabon	1000	59.93	86.9	5.5	7.2
	Ghana	1000	74.50	52.7	30.0	16.6
	Guinea	1005	62.39	91.7	3.5	3.7
	Ivory Coast	1021	67.78	85.1	7.4	6.2
	Kenya	1008	67.16	59.8	34.0	5.8
	Mali	1000	49.47	90.0	4.6	3.1
	Mauritius	1000	77.63	84.1	13.6	2.1
	Namibia	1000	63.37	61.4	29.5	6.8
	Nigeria	1004	71.31	51.5	26.3	21.4
	Senegal	1000	79.37	84.4	6.9	7.7
	South Africa	1019	69.61	48.3	16.0	35.4
	Tanzania	1001	76.46	93.6	4.2	2.1
	Uganda	1016	63.55	74.1	14.8	10.7
	Zambia	1026	64.94	66.0	29.6	4.1
	Zimbabwe	1004	55.11	64.3	25.4	10.2

Note. The type of life question also had another option “Neither”. All questions had an “I don’t know” option.

Table S2

Country Ranking for Mental Balance

Country	Mental Balance
1. Finland	92.80
2. Norway	91.00
3. Netherlands	90.66
4. Iceland	88.62
5. Sweden	88.47
6. Slovenia	88.15
7. Portugal	88.06
8. Denmark	88.00
9. Taiwan	87.33
10. New Zealand	87.03
11. Romania	86.70
12. El Salvador	86.70
13. Switzerland	86.23
14. Nicaragua	86.03
15. Malta	85.98
16. Mexico	85.38
17. Slovakia	84.68
18. Paraguay	84.47
19. China	84.45
20. Uruguay	84.38
21. Hungary	83.85
22. Canada	83.83
23. Croatia	83.73
24. Uzbekistan	83.57
25. Germany	83.30
26. Australia	83.12
27. Saudi Arabia	82.97
28. United States	82.95
29. Czech Republic	82.90
30. Indonesia	82.89
31. Costa Rica	82.80
32. Vietnam	82.67
33. Kosovo	82.43
34. Bahrain	82.42
35. Austria	82.17
36. Bosnia Herzegovina	81.95
37. Bolivia	81.85
38. Dominican Republic	81.83
39. Chile	81.80
40. Philippines	81.53
41. Kyrgyzstan	81.37
42. Argentina	80.97
43. Cyprus	80.93

44. Colombia	80.30
45. Latvia	80.25
46. Bangladesh	80.16
47. France	79.63
48. Ecuador	79.50
49. Ireland	79.50
50. Senegal	79.37
51. Malaysia	79.27
52. Montenegro	79.02
53. Italy	78.93
54. Lithuania	78.68
55. United Arab Emirates	78.34
56. Brazil	78.28
57. Moldova	78.13
58. Spain	78.03
59. Kazakhstan	77.97
60. Estonia	77.87
61. North Macedonia	77.83
62. Mauritius	77.63
63. Belgium	77.38
64. Sri Lanka	76.83
65. Jamaica	76.76
66. Thailand	76.70
67. United Kingdom	76.63
68. Tanzania	76.46
69. Bulgaria	76.43
70. Serbia	76.35
71. Japan	76.02
72. Poland	75.87
73. Greece	75.68
74. Peru	75.55
75. Mongolia	75.53
76. Myanmar	75.17
77. Albania	74.90
78. Laos	74.87
79. Ghana	74.50
80. Ethiopia	74.44
81. Hong Kong	73.30
82. Egypt	72.89
83. Ukraine	72.26
84. Jordan	72.04
85. Nigeria	71.31
86. Venezuela	71.17
87. Russia	70.90
88. Tajikistan	70.07
89. Algeria	69.95
90. South Africa	69.61

91. South Korea	69.25
92. India	68.99
93. Ivory Coast	67.78
94. Tunisia	67.50
95. Georgia	67.20
96. Kenya	67.16
97. Nepal	66.53
98. Iraq	66.20
99. Burkina Faso	65.94
100. Cambodia	65.34
101. Zambia	64.94
102. Morocco	64.21
103. Uganda	63.55
104. Namibia	63.37
105. Israel	63.20
106. Guinea	62.39
107. Turkey	61.70
108. Cameroon	60.53
109. Benin	60.52
110. Gabon	59.93
111. Pakistan	59.80
112. Iran	59.20
113. Zimbabwe	55.11
114. Congo Brazzaville	53.46
115. Mali	49.47
116. Lebanon	47.65

Table S3

Country Ranking for Preference for Calm Life

Country	Preference for calm life
1. Congo Brazzaville	96.7
2. Tanzania	93.6
3. Cameroon	92.3
4. Guinea	91.7
5. Hong Kong	91.0
6. Burkina Faso	90.2
7. Mali	90.0
8. Ethiopia	89.7
9. Nicaragua	88.4
10. Dominican Republic	87.8
11. Slovenia	87.5
12. Gabon	86.9
13. Myanmar	86.4
14. Finland	85.7
15. Japan	85.4
16. El Salvador	85.3
17. Iraq	85.2
18. Ivory Coast	85.1
19. Morocco	84.8
20. Egypt	84.5
21. Senegal	84.4
22. Jordan	84.3
23. Colombia	84.2
24. Mauritius	84.1
25. Ecuador	83.5
26. Iran	83.4
27. Sri Lanka	83.2
28. Costa Rica	82.6
29. China	82.5
30. Tajikistan	81.3
31. Taiwan	81.1
32. Brazil	80.5
33. Mongolia	80.5
34. Peru	80.4
35. Venezuela	80.4
36. Albania	80.4
37. North Macedonia	79.5
38. Uruguay	79.3
39. Mexico	79.3
40. Jamaica	78.9
41. Nepal	78.8
42. Australia	78.7

43. Benin	78.0
44. Malta	77.7
45. Thailand	77.5
46. Tunisia	77.5
47. Bosnia Herzegovina	77.4
48. Bolivia	77.1
49. Kosovo	77.1
50. Chile	77.0
51. United States	76.7
52. Saudi Arabia	75.8
53. Slovakia	75.6
54. New Zealand	75.5
55. Moldova	74.8
56. Russia	74.7
57. Bahrain	74.5
58. South Korea	74.4
59. Cyprus	74.3
60. Canada	74.3
61. Poland	74.2
62. Uganda	74.1
63. Austria	74.0
64. Paraguay	73.0
65. Argentina	72.7
66. Romania	72.3
67. Montenegro	72.0
68. Spain	71.9
69. Ireland	71.4
70. France	71.3
71. Portugal	71.2
72. Indonesia	70.2
73. Lebanon	69.8
74. Malaysia	69.6
75. Serbia	69.5
76. Netherlands	69.3
77. Hungary	68.7
78. Greece	68.4
79. Switzerland	67.7
80. Sweden	67.6
81. Croatia	66.9
82. Germany	66.5
83. Zambia	66.0
84. Philippines	65.7
85. Bangladesh	65.5
86. Czech Republic	65.4
87. Italy	65.1
88. Turkey	64.9
89. United Kingdom	64.5

90. Zimbabwe	64.3
91. Belgium	64.0
92. Bulgaria	62.8
93. Norway	61.5
94. Namibia	61.4
95. Latvia	60.9
96. Kenya	59.8
97. Uzbekistan	59.5
98. Kazakhstan	58.9
99. United Arab Emirates	58.0
100. Pakistan	57.9
101. Estonia	56.8
102. Ukraine	55.9
103. Iceland	55.1
104. Algeria	54.8
105. Ghana	52.7
106. Denmark	52.1
107. Nigeria	51.5
108. Israel	51.5
109. Laos	50.6
110. Kyrgyzstan	48.3
111. South Africa	48.3
112. Lithuania	48.1
113. Cambodia	47.8
114. India	47.1
115. Georgia	36.0
116. Vietnam	31.4
